# A simple method for the application of exogenous phytohormones to the grass leaf base protodermal zone to improve grass leaf epidermis development research

**DOI:** 10.1186/s13007-021-00828-0

**Published:** 2021-12-13

**Authors:** Jieping Li, Xinlei Feng, Jinjin Xie

**Affiliations:** College of Agriculture, School of Life Science, State Key Laboratory of Cotton Biology/State Key Laboratory of Crop Stress Adaptation and Improvement, Henan University, Kaifeng, 475004 China

**Keywords:** Stomatal development, Maize and wheat, Phytohormone, Leaf protoderm

## Abstract

**Background:**

The leaf epidermis functions to prevent the loss of water and reduce gas exchange. As an interface between the plant and its external environment, it helps prevent damage, making it an attractive system for studying cell fate and development. In monocotyledons, the leaf epidermis grows from the basal meristem that contains protodermal cells. Leaf protoderm zone is covered by the leaf sheath or coleoptile in maize and wheat, preventing traditional exogenous phytohormone application methods, such as directly spraying on the leaf surface or indirectly via culture media, from reaching the protoderm areas directly. The lack of a suitable application method limits research on the effect of phytohormone on the development of grass epidermis.

**Results:**

Here, we describe a direct and straightforward method to apply exogenous phytohormones to the leaf protoderms of maize and wheat. We used the auxin analogs 2,4-D and cytokinin analogs 6-BA to test the system. After 2,4-D treatment, the asymmetrical division events and initial stomata development were decreased, and the subsidiary cells were induced in maize, the number of GMC (guard mother cell), SMC (subsidiary mother cell) and young stomata were increased in wheat, and the size of the epidermal cells increased after 6-BA treatment in maize. Thus, the method is suitable for the application of phytohormone to the grass leaf protodermal areas.

**Conclusions:**

The method to apply hormones to the mesocotyls of maize and wheat seedlings is simple and direct. Only a small amount of externally applied substances are needed to complete the procedure in this method. The entire experimental process lasts for ten days generally, and it is easy to evaluate the phytohormones’ effect on the epidermis development**.**

**Supplementary Information:**

The online version contains supplementary material available at 10.1186/s13007-021-00828-0.

## Background

The leaf epidermis is the outer layer of leaves, with multiple functions such as preventing water loss [1]_,_ regulating gas exchange and secreting metabolic compounds. In grasses, such as maize and wheat, differentiated cell types in the leaf epidermis are epidermal cells, guard cells, subsidiary cells, and trichomes. The grass stomata are small pores, which are distributed on the upper and lower epidermis. It consist of two dumbbell-shaped guard cells with two subsidiary cells on each side [2, 3], playing a role in photosynthetic gas exchange and transpiration [4–6]_._ Many phytohormones have demonstrated an involvement in the development of stomata in dicotyledons (hereafter dicot). For example, auxin negatively regulates stomatal development [7–11]. The decreased auxin levels in the smaller daughter cell improve the equal division of the guard mother cell to produce two guard cells. On the other hand, cytokinin increases the number of stomata per leaf area in tomato, and brassinosteroid inhibits stomatal development [12–15].

The grasses are essential plant species for food, fuel, and environmental protection. Therefore, it is crucial to understand the developmental mechanisms of the leaf epidermis in grasses. Nevertheless, understanding of the effects of phytohormones on the development of leaf epidermis in grasses is limited. For maize, the exogenous addition of indole-3-acetic acid (IAA) and 1-napthaleneacetic acid (NAA) promoted the establishment of subsidiary mother cell (SMC) polarity and the formation of subsidiary cells [16]. However, the molecular mechanism of the phytohormone’s effect on monocotyledonous (hereafter monocot) leaf epidermis development is rarely elucidated. One reason is that the development and distribution of leaf epidermal cells in monocots and dicots are very different. In dicots, stem cell-like stomatal precursors are scattered in the leaf and distributed throughout the leaves, dividing and promoting these plants’ typical “broad-leaf” or radial growth characteristic [17]. In monocots, the basal meristem region is located at the leaf base. There are two zones above the meristem, the zone of initial stomatal origin and the zone of stomatal differentiation [18]. The cells below basal meristem intercalary growth make the upward cells move out of the meristematic region. Thus, epidermal cells pass through different stages of development and maturation. Maize leaf epidermal cells are typically rectangular and occur in files parallel to the leaf venation, just like rice, wheat [19, 20]. Therefore, the traditional method of exogenously spraying phytohormones, widely used in dicots to study the effect on epidermis cell development, is not suitable for monocots.

The protoderm in maize and wheat are under the leaf sheath, where there is no simple application method to deliver exogenous phytohormone [21]. Traditional methods typically add phytohormone to a culture media, such as MS medium or nutrient solution, or directly spray it on leaves. However, the operational procedure is very complicated and not direct enough [22, 23]. This study outlines a detailed method to apply phytohormone to maize and wheat seedlings to survey the effects of hormones on maize and wheat leaf epidermis development. The entire experimental process generally takes ten days, and the method is simple, allowing efficient investigation of the effect of phytohormones on epidermis development. Auxin analog 2,4-D and cytokinin analog 6-BA were used to test the system, and exogenous application of 2,4-D inhibited cell division in leaf meristematic zone and reduced guard mother cell number in maize and increased the GMC, SMC, and young stomata in wheat. In addition, 6-BA induced the enlargement of epidermal cells in maize seedlings.

## Results

### Stomatal development events in grass

**Fig. 1:**
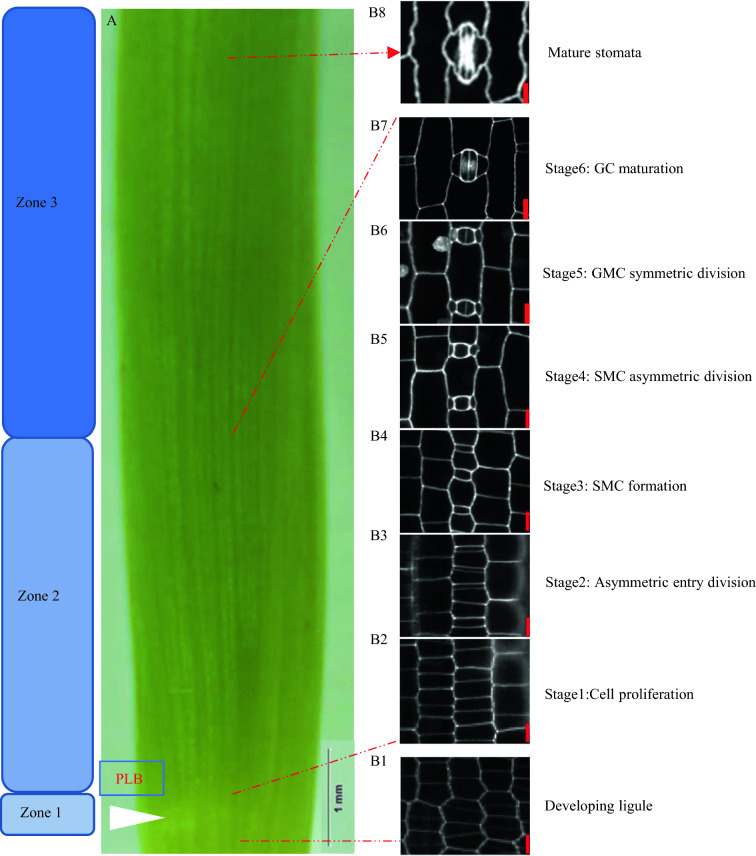
The development process of stomata in maize. **A** Zone 1 at the leaf base contains the developing ligule; Zone 2 contains the sequence series events of stomata development; Zone 3 is mature leaves fully expanded with mature stomata. **B1** The white arrow indicates the preligule band (PLB), and **B2** defines the area of stomatal lineage cell proliferation. **B3** Small guard mother cells (GMCs) were generated by asymmetric division, and **B4** expansion of the GMC induced the formation of the subsidiary mother cells (SMCs). **B5** SMCs formation via asymmetric divisions and maturation. **B6** GMCs divide symmetrically produce immature guard cells (GCs). **B7**, **B8** Young guard cell expansion and elongation to form the guard cell complex. Confocal images of propidium iodide stains were taken from the leaf base 2 of 6 days after germination of maize seedlings (B73). Red scale bar = 10 μm

The development of maize/wheat stomatal complexes followed sequence events occurring in the leaf basal meristem region. The series events begin with cell proliferation of meristematic cells, which were found in the basal meristem region beside the preligule band (PLB) (Fig. 1A, B1) [24]. The remaining series of events occurred in the stomata’s initial origin zone and the zone of stomatal differentiation, which are two consecutive zones above the meristem (Fig. 1A).

Stomatal development in grass can be divided into six consecutive stages (Fig. 1B2–B7) [25, 26]. It contains three asymmetrical divisions and one symmetrical division process. Precursor cells, particularly files at the distal end of the meristem region near the leaf base, proliferate (Stage 1, Fig. 1B2). Stomata from the first asymmetrical division produced the guard mother cell (GMC), which separated into a small rectangular cell toward the leaf tip (Stage 2, Fig. 1B3). Subsequently, the GMC asymmetrical divisions induced a pair of subsidiary cells (Stage 3 and 4, Fig. 1B4, B5). Finally, the GMC was then symmetrical divided into two immature guard cells (GC) (Stage 5, Fig. 1B6). Four especially cells mature and expand to form a mature stomatal complex (Stage 6, Fig. 1B7, B8).

Leaf tissue was extracted from 10 days old maize seedling, and its second leaf was emerging from the whorl (Fig. 8D). The coleoptile and first leaf were removed to expose developing tissue at the bases of the second leaf; three zones at basal leaf areas were excised for analysis based on developmental stages (Fig. 1A). Zone 1 is located about 80 μm of the base, containing a preligular band (PLB) structure, mainly composed of dividing cells with primarily an isodiametric shape. Zone 2 is between 100 μm to 5500 μm from the PLB and contains a cell differentiation zone. Cells in this zone vary in size and shape. It contains the pathway for stomatal development. The length of all cells in the asymmetric entry division stage is about 350 μm, and the SMC formation stage is about 400 μm, the SMC asymmetric division is about 1600 μm, and the GMC symmetric division stage is 2700 μm. Zone 3 is about 5500 μm away from the ligule, contains cells which expand into mature stomata (Additional file 1: Fig. S1).

### Exogenous application of 0.4 mg/ml 2,4-D repress cell division in maize leaf meristem zone, reduced the number of GMCs and made the ligule disappear; 0.04 mg/ml 2,4-D increased the number of GMCs, SMCs and young stomata in wheat

**Fig. 2:**
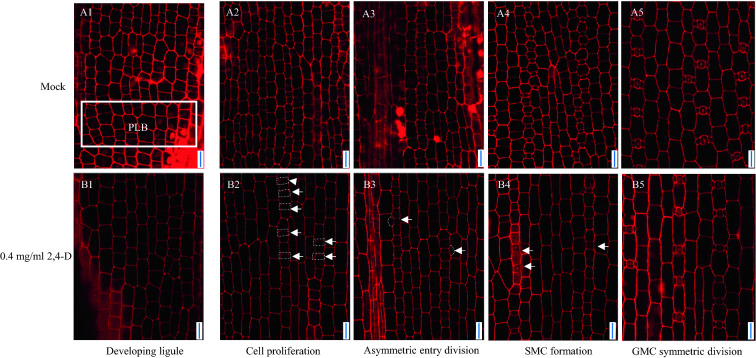
Confocal images of maize leaf protoderm treated with 2,4-D (0.4 mg/ml) and a mock treatment group. **A1–A5** Mock treatment group, and **B1–B5** 0.4 mg/ml 2,4-D treatment group. **A1** The ligule is indicated by a white solid box. **B1** The ligule disappeared at the leaf base. **B2** The number of GMC at the asymmetric entry division stage decreased, and **B3** the number of young stomata at the SMC asymmetric division stage decreased. **B4** Abnormal stomata complexes formed at the SMC formation stage in the 0.4 mg/ml treatment group. All confocal images were stained by propidium iodide. Scale bar = 20 μm

The application of 2,4-D in this work influenced the growth of leaves in maize seedlings. It mainly made the ligule disappear and reduced the GMC formation. As a result, in the protodermal areas at the leaf base, the number of GMC and epidermal cells were reduced, and the length and width of the epidermal cell increased compared with the mock treatment at the asymmetric enter division stage. In addition, some young subsidiary cells were induced by GMC, which were closer to the leaf base than the control leaves (Fig. 2B3). In general, these data indicated that 2,4-D inhibited the development of the ligule, reduced cell entry into the first asymmetric division, and suppressed the symmetric division.

A unique linear band, located at the leaf base, will develop into the ligule and auricle, named the preligule band (PLB). The cells in PLB are smaller and perpendicular to the proximal–distal axis of the developing leaf (Fig. 2A1, B1). The cells in PLB periclinal divide and develop into the ligule, which is parallel to the leaf surface [27, 28]. In the developing ligule stage, the PLB disappeared at 48 h after 2,4-D treatment. Furthermore, the frequency of smaller cells, characteristic of the PLB, was reduced in this zone, and the total number of cells in this zone also decreased significantly (Fig. 2B1).

**Fig. 3:**
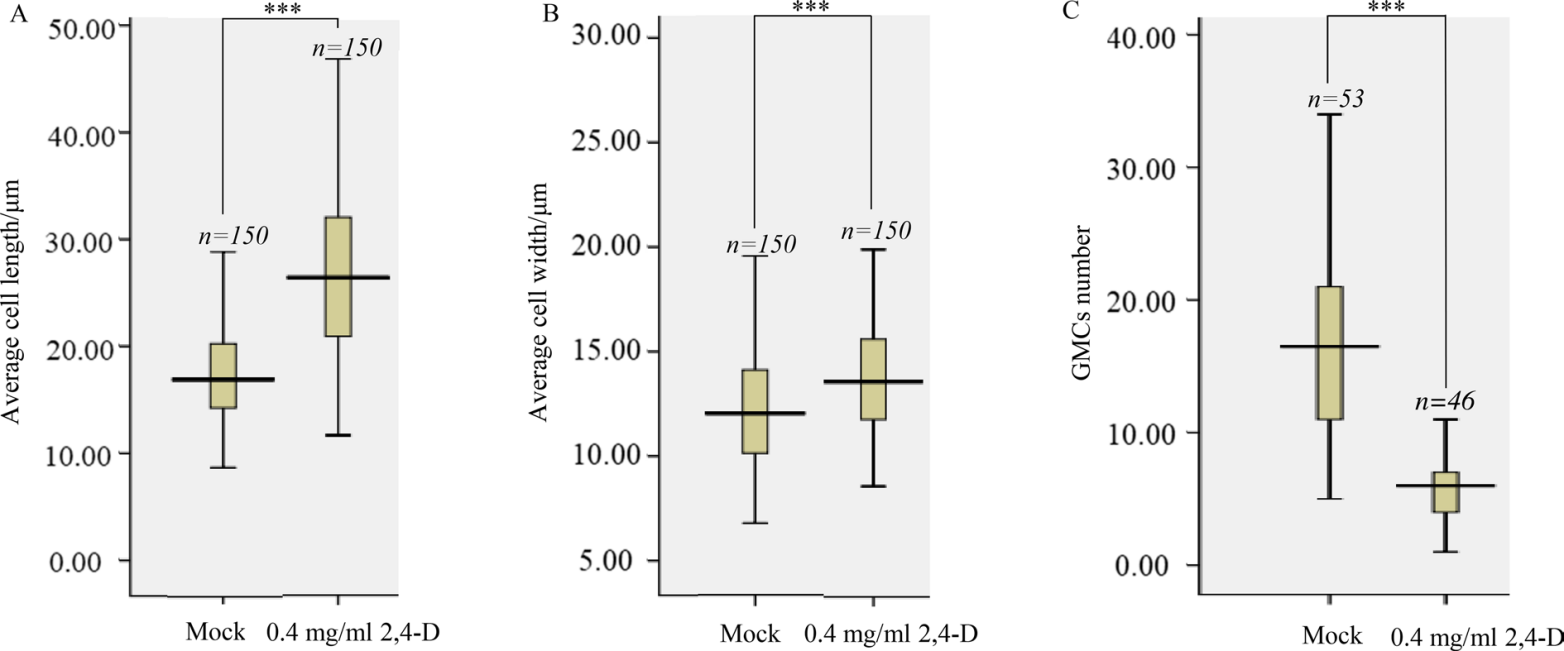
Characteristics of the cells at areas of maize leaf protoderm after 2,4-D treatment. **A** The average cell length at the cell proliferation stage after 2,4-D (0.4 mg/ml) treatment was significantly larger than the mock. Bars indicate mean (± SD) (n = 150). **B** The average cell width at the cell proliferation stage after 2,4-D (0.4 mg/ml) treatment was significantly larger than the mock. (C) The GMCs number was repressed after 2,4-D (0.4 mg/ml) treatment. Bars indicate mean (± SD) (n = 46). *** indicates significant difference within each group (i.e., p < 0.001) using the Student’s t-test

In the cell proliferation stage, the number of cells per unit area was less than the mock treatment group (Fig. 2B2). Moreover, the width and length of cells in this zone were larger than the mock treatment group. For example, the average length of cells was 26.77 μm after 0.4 mg/ml 2,4-D treatment, which was significantly longer than the mock with a value of 17.47 μm (Fig. 5A). Furthermore, the average width of cells in the 2,4-D treatment group (13.91 μm) was also significantly larger than the mock (12.19 μm) (Fig. 3B).

In the asymmetric entry division stage, the number of GMCs in the 2,4-D treatment group was less than that of the mock, and the symmetric division was also reduced compared to the mock. The GMCs density was 5.6 per 160 × 160 μm^2^ area leaf after 0.4 mg/ml 2,4-D treatment, and it was 16.6 in the mock treatment (Fig. 2B3, 3C). This result indicated that the first asymmetric division for GMCs was suppressed by 2,4-D. The events of periclinal and perpendicular divisions were also reduced, which means that 2,4-D suppressed asymmetric and symmetric division events. The number of cells in the 2,4-D treatment group was less than the mock group, possibly caused by the decrease in cell division. Should a decrease in cell division be the case, it may have also been the cause for the length and width to increase compared with the mock. Likewise, this may cause the total cell number to be less than the mock. In addition, some GMCs induce SMCs at this stage; the young stomata complex form closer to the leaf base.

Some GMCs induced SMCs to produce abnormal stomata complexes at the SMC formation stage, including two young subsidiary cells (YSCs) in the 2,4-D treatment group (Fig. 2B4). Furthermore, the stomata complex in the mock group included mature GMC flanked by two nascent SMCs (Fig. 2A4).

In the SMC asymmetric division stage, the number of cells and stomata in the 2,4-D treatment group was similar to the mock treatment group (Fig. 2B5). It indicated that the 2,4-D does not affect stomatal development at this stage, possibly because the initiation process of stomata in this stage is completed before the 2,4-D treatment. It indicates that the initial process of stomata initiation may be influenced by 2,4-D, but when the initial process was completed, even under the condition of 2,4-D treatment, GMC will develop into mature stomata complexes.

**Fig. 4:**
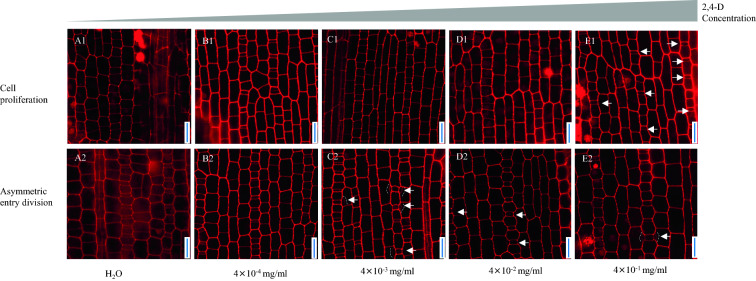
Confocal images of maize leaf protoderm treated with different concentrations of 2,4-D (4 × 10^–4^ ~ 4 × 10^–1^ mg/ml). The top row **A1**–**E1** represents the cell proliferation stages of the leaf protoderm’s treatment area with different concentrations of 2,4-D (4 × 10^–4^ ~ 4 × 10^–1^ mg/ml). The bottom row **A2**–**E2** is the asymmetric entry division stage of the leaf protoderm area of treatment with different concentrations of 2,4-D (4 × 10^–4^ ~ 4 × 10^–1^ mg/ml). All confocal images were stained by propidium iodide. Scale bar = 20 μm

The minimum concentration of 2,4-D that influences subsidiary cell formation in maize is 4 × 10^–3^ mg/ml. At the asymmetric entry division stage, many young subsidiary cells in the 2,4-D treatment group were induced by GMCs at a concentration of 4 × 10^–3^ mg/ml (Fig. 4C2). Moreover, many GMCs induced subsidiary cells at 4 × 10^–2^ mg/ml and 4 × 10^–1^ mg/ml (Fig. 4D2, E2). The young GMC at the asymmetric entry division stage did not induce the subsidiary cells under 2,4-D treatment conditions at a concentration of 4 × 10^–4^ mg/ml (Fig. 4B2), just like the young GMC in the mock treatment group. This result indicates that for the 2,4-D treatment in maize, the minimum concentration that affects the subsidiary cell formation is 4 × 10^–3^ mg/ml.

**Fig. 5:**
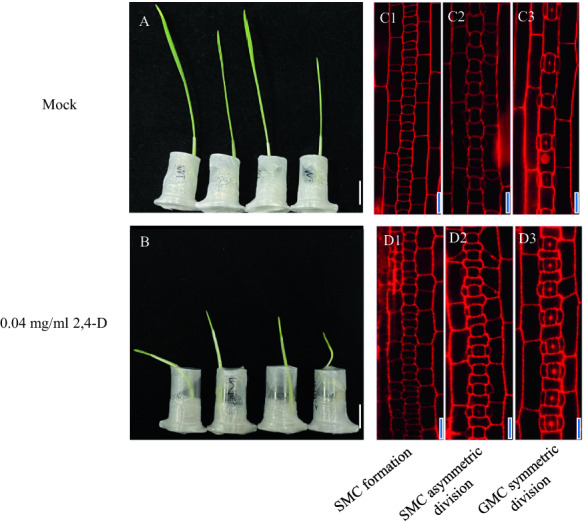
The phenotype of wheat seedlings after treatment with 0.04 mg/ml 2,4-D. **A** Mock treatment of wheat seedlings, and 0.04 mg/ml 2,4-D after 36 h. **B** The density of GMC at SMC formation stage, GMC and SMC at SMC asymmetric division stage, young stomata at GMC symmetric division stage were increased after 0.04 mg/ml 2,4-D treatment. **C1**–**C3** The mock treatment group, and **D1**–**D3** the 0.04 mg/ml 2,4-D treatment group. **D1** The density of GMC at SMC formation stage, **D2** GMC and SMC at SMC asymmetric division stage, and **D3** young stomata at GMC symmetric division stage were increased after 0.04 mg/ml 2,4-D treatment. The white bar in A and B represents 1 cm. Scale bar = 20 μm

For wheat variety Chinese Spring, after treatment with 0.04 mg/ml 2,4-D for about 36 h, compared with mock treatment, the seedling height was significantly reduced (Fig. 5A). In addition, the density of GMC in the SMC formation stage, GMC and SMC in SMC asymmetric division stage, young stomata in the GMC symmetric division stage (Fig. 5B) were increased after 0.04 mg/ml 2,4-D treatment.

### Exogenous application of the 6-BA enlarged the epidermis cells’ size and reduced the stomata density of maize seedlings

**Fig. 6:**
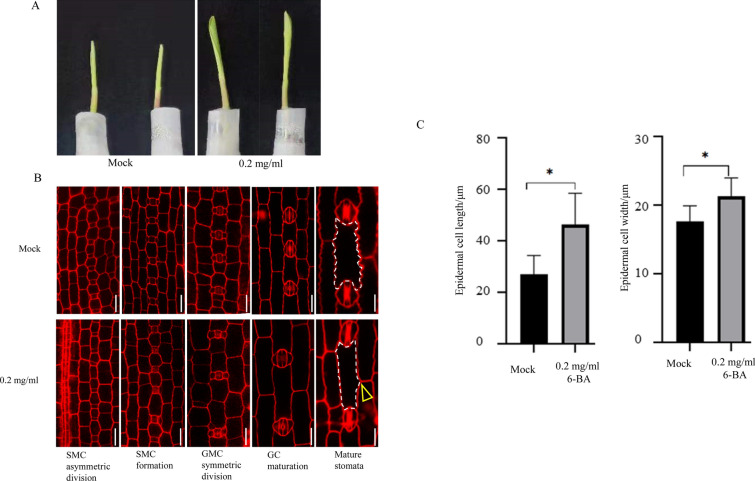
The phenotype of maize seedlings after 6-BA treatment. **A** Maize seedlings representing the mock treatment and 0.2 mg/ml 6-BA after 36 h. **B** The difference of developed stomata between the mock group and 0.2 mg/ml 6-BA treatment group. All confocal images were stained by propidium iodide. Scale bar = 20 μm. **C** The difference between epidermal cells length and width between mock group and 0.2 mg/ml 6-BA treatment group

After 0.2 mg/ml 6-BA treatment, the height of maize seedlings increased, and its first leaves were unfolded and tightly bound compared to the mock-treated group (Fig. 6A). The confocal images of the seedling’s leaf protoderm showed that the cells between two GMCs in the 0.2 mg/ml 6-BA treatment group were longer than the mock-treated group at the SMC formation stage. Furthermore, the length of cells between the young stomata complex in the 0.2 mg/ml 6-BA treatment group became longer than the mock-treatment group at the GMC symmetric division and GC maturation stages. This result indicates that the stomata numbers in the stomatal development cell lines decreased after 6-BA treatment. For other epidermal cells, the cell width in the 6-BA treatment group was significantly wider than that of the mock-treated group (Fig. 6B). At the mature stomata stage, the average length of epidermal cells in the 6-BA treatment group was about 49.26 μm; the width was about 21.29 μm. In contrast, in the mock-treated group, the average length of epidermal cells was about 27.01 μm, with a width of about 17.64 μm, and there was a significant difference between the 6-BA exogenous applications group and the mock-treatment group (Fig. 6C). Furthermore, at the mature stomata stage, the epidermal cell shape became irregular after the 6-BA treatment, which was different from the square shape of the epidermis cells in the mock-treatment group (shown by yellow arrow in Fig. 6B). In particular, the shape of the epidermal cells between the stomata complexes became rhomboid.

In summary, after 6-BA treatment, the epidermal cells became longer and wider than the mock-treatment group. Further, the density of stomata decreased, and the shape of the epidermal cell, especially the cells between stomata complex cells, became irregularly shaped, similar to a diamond.

## Discussion

In grass, the growth process of leaves is linear. Cell division occurs at the leaf basal zone, followed by cell expansion and maturity at the leaf tip [29]. For a single layer of cells, the epidermis can be treated as a one-dimensional structure. Cells in the grass leaf epidermis divide and expand along the same axis in unison. The cell division process provides additional underlying new cells. After division, cells expand to increase the cellular volume [29, 30]. The base zone with dividing cells in the leaf epidermis is consistently referred to as the protoderm [31]. Thus, the leaf protoderm plays a crucial role in the development of the leaf epidermis. Moreover, it represents an ideal model system for quantitative research on cell division and spatial expansion patterns. It is also a valuable and rudimentary research system in plant developmental biology.

Phytohormones are small molecular substances with biological activity, such as small molecular peptides, regulating plants’ development, including leaf protoderm. The application of these regulatory substances is an effective method to study the molecular mechanism of protoderm development. However, the protoderm of monocot plants is covered by leaf sheaths. The traditional method of spraying on the surface of leaves can affect leaf development, such as spraying GA on the leaves surface of dwarf mutants can rescue a phenotype in mutant plants [32]. Spraying or immersing the synthetic 6-benzylaminopurine (6-BA) or ABA to tomato is suitable for dicots, in which meristemoids cover the whole leaf [12]. However, the spraying operation is not targeted and is generally used for long-term phenotyping observation. Furthermore, the precise quantification and observation time of externally applied substances is unclear, and a large amount of externally applied substances is required, which may be costly and inefficient. Another widely used method for exogenous phytohormone application is the addition of phytohormones into the culture medium. Many phytohormones are used in this method, such as brassinosteroid [14] and auxin [7]. The root system absorbs phytohormone into the plant body, and the root system is affected first. Abnormal roots will affect the absorption of nutrients and water and may affect the plants’ normal (wild type) growth. Thus, the protoderm’s abnormal development will not be due to the direct interaction of externally applied substances, only physiological issues arising from other regions of the plant. Moreover, as an aseptic operation is always required, the process can be complicated, and the experimental cycle is long.

In the method reported in this paper, phytohormones were applied externally to the grass mesocotyls, and the root system was removed to avoid its physiological effects. The adsorption effect of absorbent cotton was used to maintain the quantitative concentration of phytohormones. Thus, it can be processed with very few reagents. The treatment time of the external application is generally 2 days. During the treatment, the externally applied hormone maintains a stable concentration, the treatment operation is simple, and the phenotypic observation method is quick and convenient. Thus, it is suitable for the study of the developmental mechanism of protoderm in grasses.

In this study, exogenous applications of 2,4-D to maize seedlings repressed cell division in the leaf meristem zone, reducing the number of GMCs and making the ligule disappear. Auxin is one of the plant developmental master hormones. It controls stem cell compartment size during stomatal development in *Arabidopsis*. The PIN-FORMED protein-mediated intercellular auxin polar transport, PIN-GFP fluorescence, is especially strong around stomatal precursor cells, meristemoids and guard mother cells. High efflux of auxin from meristemoids, mediated by PIN3, is required for fate transition from the meristemoids to guard mother cells. Some PIN mutants show defective stomata patterns. Cell fate transfer during the stomata development process probably requires PIN-mediated auxin transport. Auxin and the auxin-responsive gene Monopteros (MP) work together to repress the expression of STOMAGEN in the mesophyll. The STOMAGEN as a mobile peptide can improve the development of stomata. 2,4-D treatment decrease STOMAGEN expression significantly in *Arabidopsis* seedlings. MP binds to the STOMAGEN promoter directly, and the genetic studies about STOMAGEN-RNAi; *arf5-1*/ + transgenic line indicated that MP regulates stomata development through repressing STOMAGEN expression [10]. In this study, 2,4-D repressed the asymmetric division at the asymmetric entry division stage in maize, but increased the number of wheat GMC, SMC, and young stomata. The main reason is the different sensibility between maize and wheat seedlings for 2,4-D treatment. In the experiment about wheat seedlings treated with different concentration 2,4-D, the height of wheat seedlings is similar with mock group at 4 × 10^–3^ mg/ml, and at 4 × 10^–2^ mg/ml and 0.4 mg/ml, the height of wheat seedlings was depressed significantly (Additional file 1: Fig. S2A). For the wheat leaf protoderm, at 4 × 10^–3^ mg/ml treatment group, the GMCs number were increased, it is increased at 4 × 10^–2^ mg/ml and 0.4 mg/ml (Additional file 1: Fig. S2B). In the experiment about maize seedlings treated with different concentration 2,4-D, when the 2,4-D’s concentration was 0.4 and 4 mg/ml, the height of maize seeding is similar with mock. When the 2,4-D’s concentration was increase to 40 mg/ml, the height of maize seeding shown a little depressed (Additional file 1: Fig. S2C). This result indicated that wheat seedlings is more sensitive than maize’s, its growth is significantly inhibited at low concentration of 2,4-D (4 × 10^–2^ mg/ml), and maize seedling required a higher concentration than 40 mg/ml to produce some inhibitory effect. The different sensibility between maize and wheat seedlings treated with 2,4-D also be proved in water culture [33]. It is worthwhile asking whether the molecular mechanism behind this physiological phenomenon is similar to *Arabidopsis*. Future works are required to further this research.

Auxin is a crucial player in the generation of subsidiary cells in maize. Spatiotemporal changes of PIN protein indicated local auxin transfer from GMCs to SMCs [16]. Combined with our experimental results, the auxin plays a vital role in establishing polarity in SMC. In maize, *ZmMUTE*, a bHLH transcription factor, plays a vital role in determining the fate of SMC. It induced SMC polarization and regulated the last symmetrical division of GMCs, resulting in two GCs. It is an essential regulator of PAN1 and PAN2. PAN2 is polarized in premitotic SMCs [34]. PAN1 and its downstream gene ROP2/9 proteins are polarized after PAN2 polarization. All of these, together with F-actin, induce nuclear migration towards the GMC proximal site and polarize SMC [34–36]. In this study, many young subsidiary cells were induced by GMC even in the asymmetric entry division stage in the 2,4-D treatment group at a concentration of 4 × 10^–3^ mg/ml, indicating that 2,4-D improves the SMC form. More work could be carried out to clarify the relationship between 2,4-D and *ZmMUTE*, *PAN1*, *PAN2*. After treatment with 2,4-D, the expression levels of these genes could be observed, and the phenotype of mutants of *pan1*, *pan2* and *ZmMUTE/BZU2.* Surveying phenotypic data, alongside expression levels after 2,4-D treatment, could reveal the mechanism of 2,4-D inducing the SMCs.

Cytokinin regulated the leaf cell expansion process [37, 40] and increased plant cell size by promoting cell wall elongation, improving turgor pressure, and increasing endoreduplication [38–40]. During plant leaf cell expansion, the cell walls undergo three essential processes: loosening, remodeling, and biosynthesis [41]. Plant cells perform faster cell elongation under acidic conditions. During these conditions, auxin stimulates the plasma membrane H^+^-ATPase proton pump activity [42, 43]. In poplar leaves, exogenous cytokinin induced the expression of EXPA3, which has the function of expanding the cell wall [44]. In *Arabidopsis*, CKX2 induces the expression of EXPA5, overexpression of CKX1 improves the leaf cell expansion [38, 45]. Maybe this is the mechanism for the exogenous application of 6-BA to increase the length of epidermal cells in this research. Excessive cytokinin increases major soluble sugars and starch, stimulating carbohydrate metabolism and energy-associated processes [38]. In addition, they increase the turgor pressure of cells, thereby relaxing the cell wall [46]. Cytokinin induced cell endoreduplication by regulating the expression of CCS52A1, which plays a role in increasing ploidy by chromosome replication without subsequent cell division, also participates in processing the cell size increase [47, 48].

## Conclusion

This paper presents a convenient and straightforward method for the exogenous application of phytohormone to the protodermal areas in grass leaves. At five days after germination, the seedlings were separately placed in a piece of self-made processing equipment, and absorbent cotton was used to maintain an external reagent for continuous supply for three days. After treatment, the cell wall was stained by PI, and the phenotype was evaluated under a confocal system. The entire experimental process generally lasted for ten days, and it is easy to evaluate the effects of the phytohormone on epidermal development. The auxin analogs 2,4-D and cytokinin analogs 6-BA were used to test the system, and in maize, 2,4-D repressed initial stomatal development. Exogenous application of 2,4-D (0.4 mg/ml) repressed the leaf meristem cell division, decreased the number of GMCs and made the ligule bands disappear. After wheat seedlings were treated with 0.04 mg/ml 2,4-D, the density of GMC, SMC and young stomata increased. After 6-BA treatment, the maize leaf epidermis cell became longer and wider, and the stomata density decreased. The results indicated that the method is suitable for applying phytohormone to leaf protodermal areas in grasses.

## Methods

### Maize seedlings germination

**Fig. 7:**
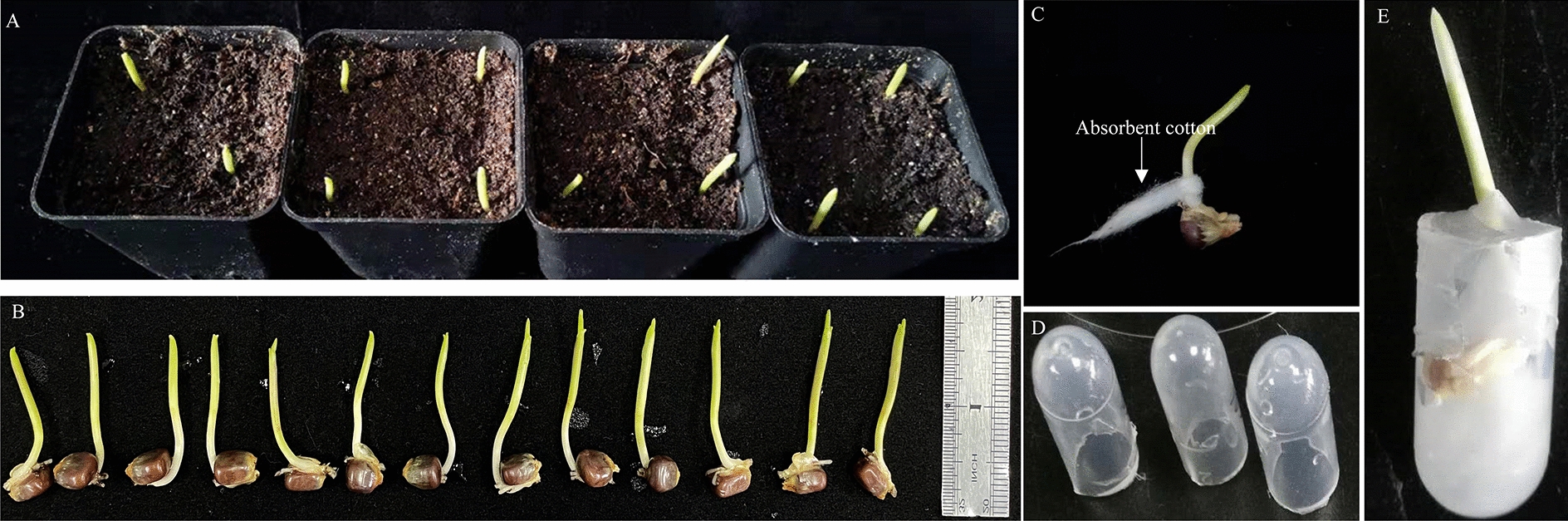
Germination of maize seedlings and exogenous application of phytohormone. **A** The germination of maize seedlings in square culture pots, and **B** subsequent primary and embryo roots removal. **C** Then, absorbent cotton saturated with hormones is wrapped around each maize seedling’s mesocotyl. **D** Handmade 2.5 ml tubes, and **E** final treatment setup

Full seeds of maize (*Zea mays* L.) inbred lines B73 or W22 and wheat variety Chinese Spring kernels, without shrinkage and mildew, were selected and planted in a sterile mixed culture matrix (vermiculite: nutrient soil: black soil, 1:1:1). The planting depth is 2 cm from the soil surface for maize and 1 cm for wheat. Four kernels were planted in a 5 × 5 × 5 cm^3^ square pot, 12 pots were placed in each 20 × 35 cm^2^ plate, and 48 seeds were planted in one plate (Fig. 7A). On the day of planting, 100 ml sterile water was added to keep the soil medium moist. The plate was kept in an incubator with the following parameters: temperature 26/23 °C, photoperiod 16/8 h light/dark cycle. Three days after planting, 100 ml sterile water was added for the second time. After four or five days, the coleoptile of maize or wheat seedlings can be seen breaking the soil surface. One week after sowing, the first leaf had just exposed the coleoptile (Fig. 7A).

### Methods for exogenous application of phytohormone

**Fig. 8:**
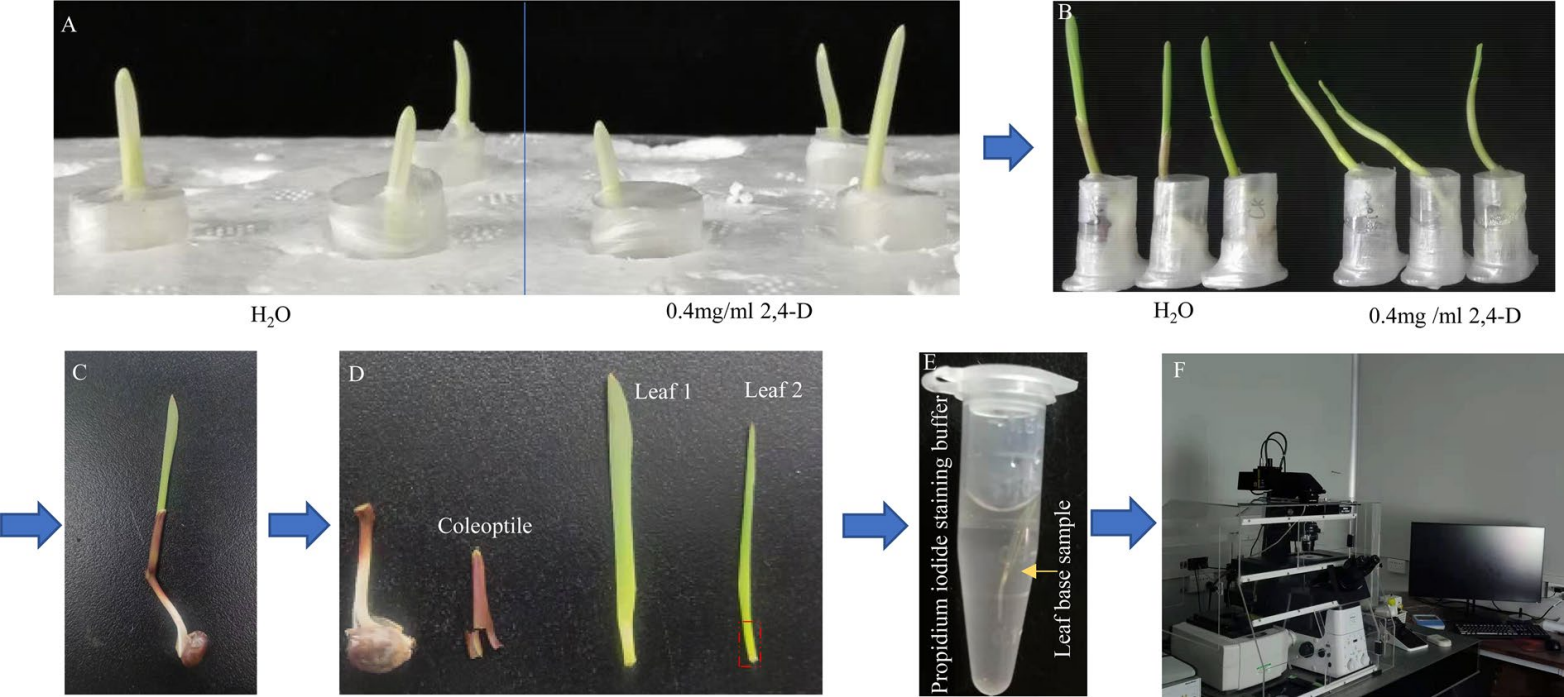
Investigation of maize seedling anatomy and phenotype. **A** Maize seedlings treated with 0.4 mg/ml 2,4-D, and **B** treatment with 0.4 mg/ml 2,4-D after 36 h. **C** Maize seedling post-treatment. **D** Anatomy of the seedings and the leaf base for collection section to be excised defined by a red dotted box. **E** The leaf base samples were stained by propidium iodide, and **F** the phenotype was investigated using a confocal microscope

Seven days after sowing, the maize coleoptiles were approx. 2 cm long, the first leaf grew out of the coleoptiles, and five days after sowing, the wheat seedlings were suitable for treatment. The uniform seedlings were selected for phytohormone treatment. Whole seedlings were removed, soil stuck to the seedlings was shaken off, and seedlings were rinsed with water. Scissors were used to cut off the primary roots and seminal roots (Fig. 7B). Seedlings were kept in moistened water-absorbent paper to prevent dehydration of the young tissues. Absorbent cotton containing external hormones was wrapped on the mesocotyls of the seedlings (Fig. 7C) and kept a part of the absorbent cotton sticking out; 400 μl treatment solvent was added to the self-made 2.5 ml centrifuge tube (Fig. 7D). The seedlings wrapped in absorbent cotton were planted in centrifuge tubes to keep the absorbent cotton in contact with the treatment solvent and ensure that external phytohormones were proximal for continuous treatment. Parafilm was used to seal the centrifuge tube to avoid volatilization (Fig. 7E, 8A), and the processing device was placed in the incubator (temperature 26/23 °C, photoperiod 16/8 h light/dark cycle) to continue the culture. An additional control experiment was performed by placing control seedlings on cotton wetted with the solvent (distilled water or dimethyl sulfoxide with a maximum concentration below 0.5% (v/v)). Experiments were repeated at least three times.

### Anatomy and phenotype investigation of leaf epidermis

After 48 h of treatment, the maize/wheat seedlings were taken out of the processing device (Fig. 8B, C, and the stems of the seedlings were dissected using forceps and scalpel blades, as well as the coleoptile and first leaf wrapped in the outer layer, was removed (Fig. 8D). Detailed dissection of the second leaf was performed, the sample containing 1 cm from the base of the stem was saved, and the sample was placed in 10 mg/ml propidium iodide (PI, Beijing Solarbio Science & Technology Co., Ltd) staining dye solution for 5 min (Fig. 8E). After dyeing, a sample was removed, and dye attached to the sample’s surface was washed off with water and placed on a glass chip. The phenotype was observed under a confocal microscope (Fig. 8F). PI is applied to stain cell walls with a fluorescence excitation maximum of 535 nm and an emission maximum of 617 nm [49]. A series of overlapping images were taken along the leaf apex by a fluorescence confocal microscope (Nikon A1, Nikon, Japan). The length and width of different types of cells were measured by ImageJ software.

## Supplementary Information

**Additional file 1:** **Figure S1.** The development pathway of stomata in the second leaf at 7 days after sowing maize seedling. (A) Large scale confocal images of maize leaf protoderm. (B-F) Series events of stomata development. **Figure S2.** The phenotype of wheat and maize seedlings treated with different concentration of 2,4-D. (A) Wheat seedlings treated with different concentration 2,4-D (4 × 10-3~4 × 10-1 mg/ml). (B) Confocal images of maize leaf protoderm treated with different concentrations of 2,4-D (4 × 10-3~4 × 10-1 mg/ml). Scale bar = 20 um. (C) Maize seedlinggs treated with different concentration 2,4-D (0.4-40 mg/ml). The white bar in A and C represents 1 cm.

## Data Availability

The datasets used and/or analysed during the current study are available from the corresponding author on reasonable request.

## Abbreviations

IAA: Indole-3-acetic acid
NAA: 1-Napthaleneacetic acid
SMC: Subsidiary mother cell
PI: Propidium iodide
GMC: Guard mother cell
GC: Guard cells
PLB: Preligular band
YSC: Young subsidiary cell
6-BA: 6-Benzylaminopurine
MP: Monopteros
GA: Gibberellic Acid
