# Supplementary material for: A simple method for the application of exogenous phytohormones to the grass leaf base protodermal zone to improve grass leaf epidermis development research
Source: Plant Methods. 2021 Dec 13;17:128. doi: 10.1186/s13007-021-00828-0 (PMC8667372; doi:10.1186/s13007-021-00828-0)
Supplement: Supplementary file 1 — Additional file 1: Figure S1. The development pathway of stomata in the second leaf at 7 days after sowing maize seedling. (A) Large scale confocal images of maize leaf protoderm. (B-F) Series events of stomata development. Figure S2. The phenotype of wheat and maize seedlings treated with different concentration of 2,4-D. (A) Wheat seedlings treated with different concentration 2,4-D (4 × 10^-3^~4 × 10-1 mg/ml). (B) Confocal images of maize leaf protoderm treated with different concentrations of 2,4-D (4 × 10^-3^~4 × 10-1 mg/ml). Scale bar = 20 um. (C) Maize seedlinggs treated with different concentration 2,4-D (0.4-40 mg/ml). The white bar in A and C represents 1 cm. [file 13007_2021_828_MOESM1_ESM.pdf]

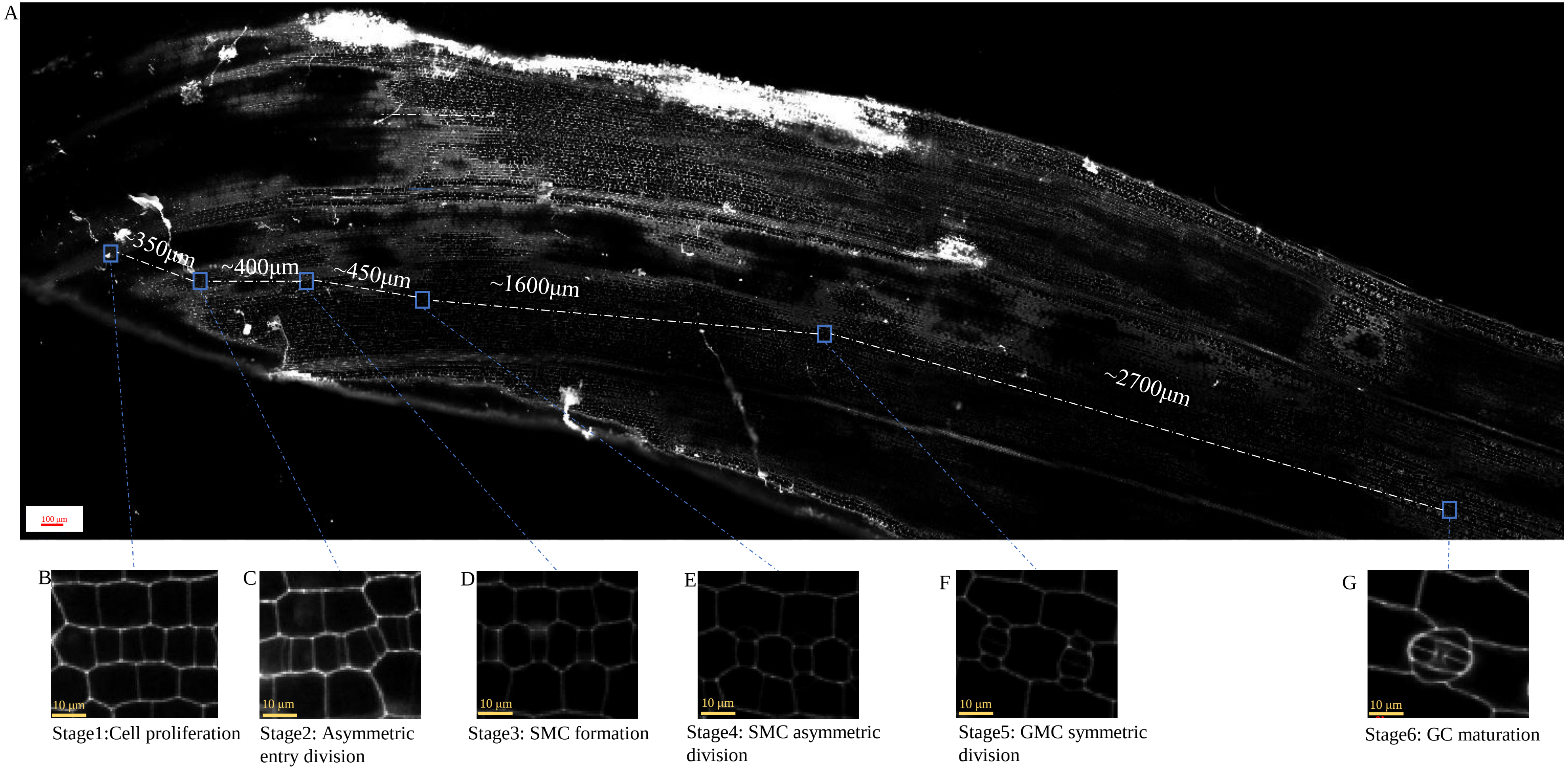

Supplementary Figure 1 The development pathway of stomata in the second leaf at 7 days after sowing maize seedling. (A) Large scale confocal images of maize leaf protoderm. (B-F) Series events of stomata development.

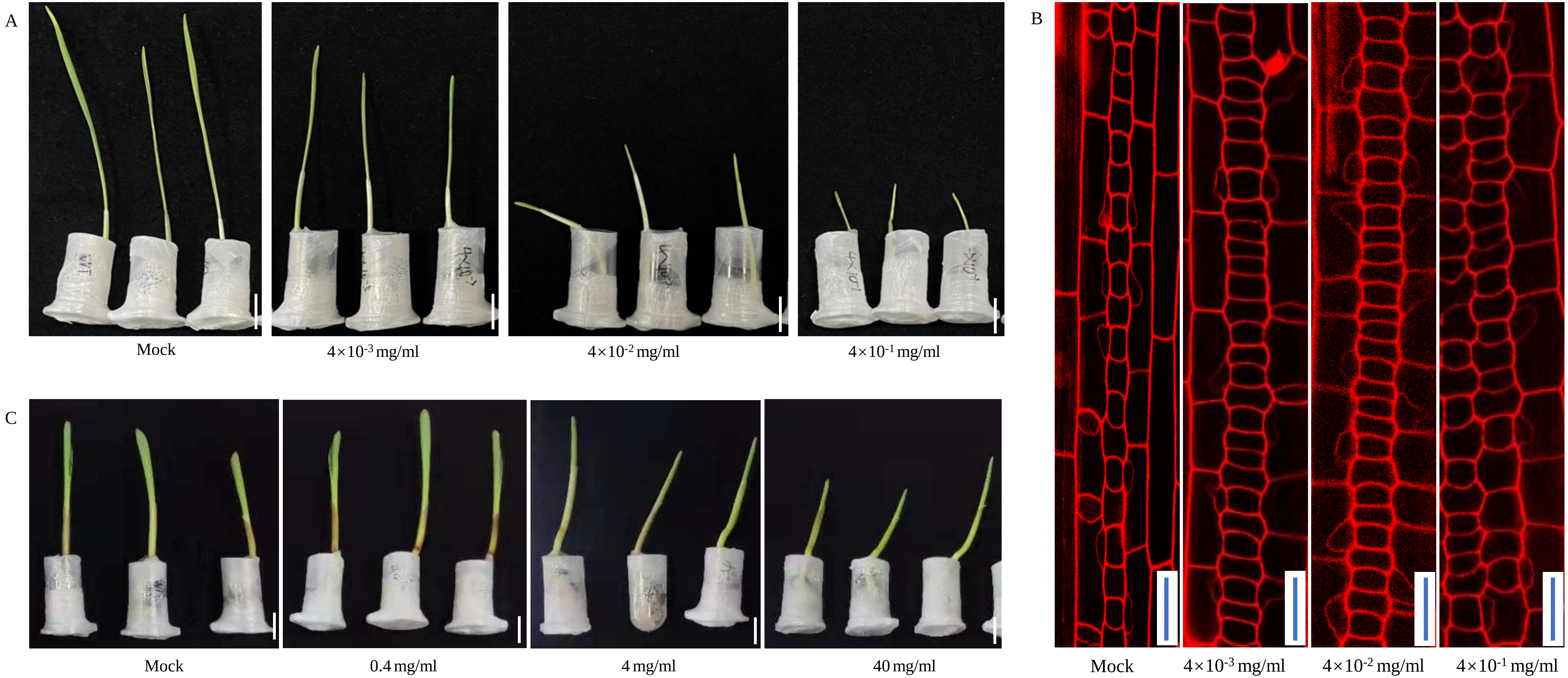

Supplementary Figure 2 The phenotype of wheat and maize seedlings treated with different concentration of 2,4-D. (A) Wheat seedlings treated with different concentration 2,4-D (4×10-3~ 4×10-1 mg/ml). (B) Confocal images of maize leaf protoderm treated with different concentrations of 2,4-D (4×10-3~ 4×10-1 mg/ml). Scale bar = 20 μm. (C) Maize seedlings treated with different concentration 2,4-D (0.4~40 mg/ml). The white bar in A and C represents 1cm.
